# Model test study of bridge pile horizontal bearing behavior under landslide deformation

**DOI:** 10.1038/s41598-024-64258-3

**Published:** 2024-07-01

**Authors:** Yan-yan Zhao, Hai-ying Fu, Tao Yang, Ming-zhe Zhou, Huan Li

**Affiliations:** School of Civil Engineering, Southwest Jiaotong University, Chengdu, 610031 China

**Keywords:** Civil engineering, Engineering

## Abstract

Pile is a common foundation on the slope, which poses a serious threat to the construction and operation if the slope deformation and causes landslide. In this study, a model device of pile foundation on landslide was independently developed by relative displacement loading between pile and soil to explore the influence of landslide deformation on pile and analysis the soil failure rule and the deformation characteristics of pile in different stages of landslide deformation, a few model tests were completed including the relative displacement between soil and pile from 1 to 17 cm, and the pile diameter and the modulus of slide bed were also considered. The results indicated that: the evolution process of landslide deformation with pile foundation on could be divided into four stages including soil compaction, cracks growth, yield stage, and failure stage; ratios of the maximum soil pressure and bending moment growth from the soil compaction stage to the cracks growth stage to the total growth in these four stages are both exceeding 60%; the soil pressure increases with the increase of pile diameter and sliding bed modulus. Therefore, it is best to effectively monitor and control the landslide in the initial soil compression stage that in soil compaction stage and methods such as increasing pile foundations or reinforcing the sliding bed can be used for protection.

## Introduction

**Figure 1:**
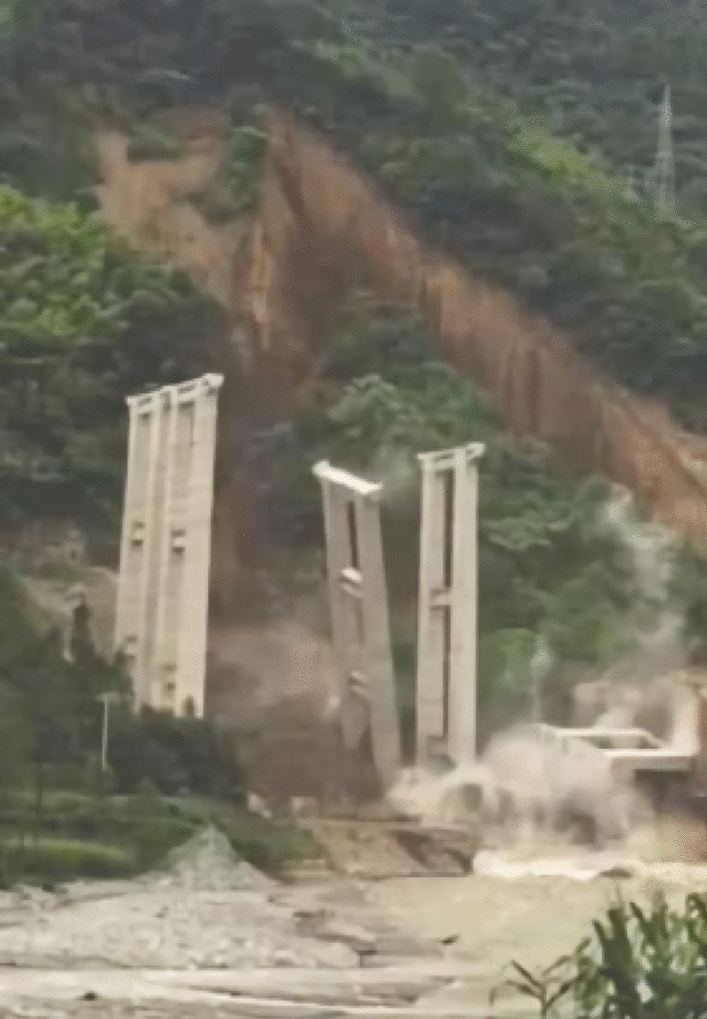
Landslide causes damage to bridge foundation ^4^.

Pile foundation has been applied in a few engineering programs such as high-speed railway, long-span bridge et al. Compared with pile mainly constructed on the level ground such as high-speed railway, bridge, and more buildings always site on the slope. In addition, before the construction of the superstructure, such as bridge, pile foundation has been on the slope under the corresponding soil pressure, in this process, once the slope deformation due to heavy rain, unloading and other factors, the soil will produce creep deformation and integral sliding, which would directly affect the bearing capacity of pile^1–3^. As shown in Fig. 1, a landslide occurred in Tianquan County, resulting in the collapse of eight pier columns of Daren Yan Bridge on Yakang Expressway in July 2016, so clear the relationship between slope deformation and pile stress is essential.

Many researches have studied the pile foundation of bridge and other transportation infrastructure, most studies focus on the pile foundation on level ground, some of them have studied the interaction between pile foundation and soil on level ground^5–7^, a few of them have researched the elastic characters of single piles and group piles using numerical analysis^8–15^. Senjuntical et al.^16^ developed a theoretical model to calculated the consolidation settlement and analyzed the axial load transfer rules of vertically loaded pile groups in multilayered poroelastic soils. Wang et al.^17^ provides a theoretical basis for reinforcement of the soil around multi-wing piles, and the limit analysis was used to determine the ultimate lateral capacity (ULC) of three- and four-wing piles in Dapeng Bay silty clay. Recently in China, more and more bridges, railways, and other transportation facilities were built in canyons, and their pile foundations are correspondingly built on slopes. Therefore, it is of great significance to consider the influence of slope deformation on pile foundations.

In many practical projects, pile foundation is often studied on the slope. For example, many people have presented different methods to calculated the soil resistance of pile by theoretical analysis^18–24^, numerical methods^25–27^, model tests^28–30^ and full-scale tests^31–33^. Some researchers have been proposed various methods to predicted the lateral resistance of piles^29,34–38^. In addition, the slope could reduce the bearing capacity of pile, which was influenced by the position of pile on slope^25,26^. Unfortunately, available reference for piles on slope with slope deformation is very limited, and few scholars have conducted in-depth research on the deformation of slope in pile foundation construction and its influence on the force of pile foundation.

According to the previous research, this paper has carried out the model test about the effect of landslide deformation on the soil pressure and bending moment of pile. Due to artificial loading or hydraulic device that are used to make deformation of landslide always takes a long time, and the labor cost is high with slow change of soil. In this study, it is considered that the deformation of soil behind the pile is entire and the resistance of soil in front of pile in slip mass are ignored, the soil would be compressed when the pile moved towards to soil behind the pile, this method is used to replace the thrust force on the pile due to soil instability. The relative distance between pile and soil including 1 to 17 cm were considered, and for further study, the pile diameter and sliding bed modulus were also changed to study the influence of landslide deformation on pile and analysis the soil failure rule and the deformation characteristics in different stages of landslide deformation.

## Model test

### Soil condition

The simulation of free segment and embedded segment materials is based on the test method of Zhu et al.^39^, and the similar model materials are mainly composed of barite powder, bentonite, land plaster, washing powder, silica sand and water. Barite powder mixed with sand are mainly used as a weighting agent to increase the internal friction angle and permeability coefficient of slip mass. Bentonite is mainly used to increase the cohesion of slip mass and reduce the permeability coefficient. Land plaster is used to increase the smoothness of slip mass materials. Washing powder plays a very good adsorption effect on water, easy to form the deformation of slip mass. The slip mass was constructed by layered filling. The mass ratio of each similar model materials is shown in Table 1.

**Table 1:** Similar model materials and mass ratio.

Types	Silica sand (%)	Bentonite (%)	Barite powder (%)	Land plaster (%)	Washing powder (%)	Water (%)
Slip mass	50	15	15	5	8	7
Slide bed	45	25	15	5	5	5

Considering the size of the model test system, this experiment used geometric similarity ratio C_L_ of 30 to design the landslide dimension. The geometry, density, and acceleration as the basic physical parameters, due to the geometric similarity ratio is 1:30, and the density and acceleration similarity ratio are both 1:1, the similarity ratio of other physical parameters could be calculated according to the Buckingham π as described in Table 2. Meanwhile, the specific parameters of soil are shown in Table 2, the specific parameters of landslide are shown in Table 3, the density (ρ), cohesion (c), internal friction angle (φ), poisson’s ratio (μ) and elastic modulus (E) of landslide have been provided.

**Table 2:** Similarity relations and ratios.

Number	Physical parameters	Symbol and relational expression	Similarity ratio
1	Length	C_L_	1:30
2	Density	C_ρ_	1:1
	Acceleration	C_a_	1:1
3	Elastic modulus	C_E_ = C_L_ C_a_ C_ρ_	1:30
4	Cohesion	C_c_ = C_E_	1:30
5	Internal friction angle	C_φ_	1:1
6	Poisson’s ratio	C_μ_	1:1

**Table 3:** Physical and mechanical parameters of landslide.

Types	Prototype					Model				
	ρ (g/cm^3^)	φ (°)	c (kPa)	μ	E (MPa)	ρ (g/cm^3^)	φ (°)	c (kPa)	μ	E (MPa)
Slip mass	1.90	30.0	25.00	0.30	80	2.01	27.55	1.932	0.31	2.6
Slide bed	2.10	–	–	0.27	400	2.05	–	–	0.32	13.00

### Model device and load system

**Figure 2:**
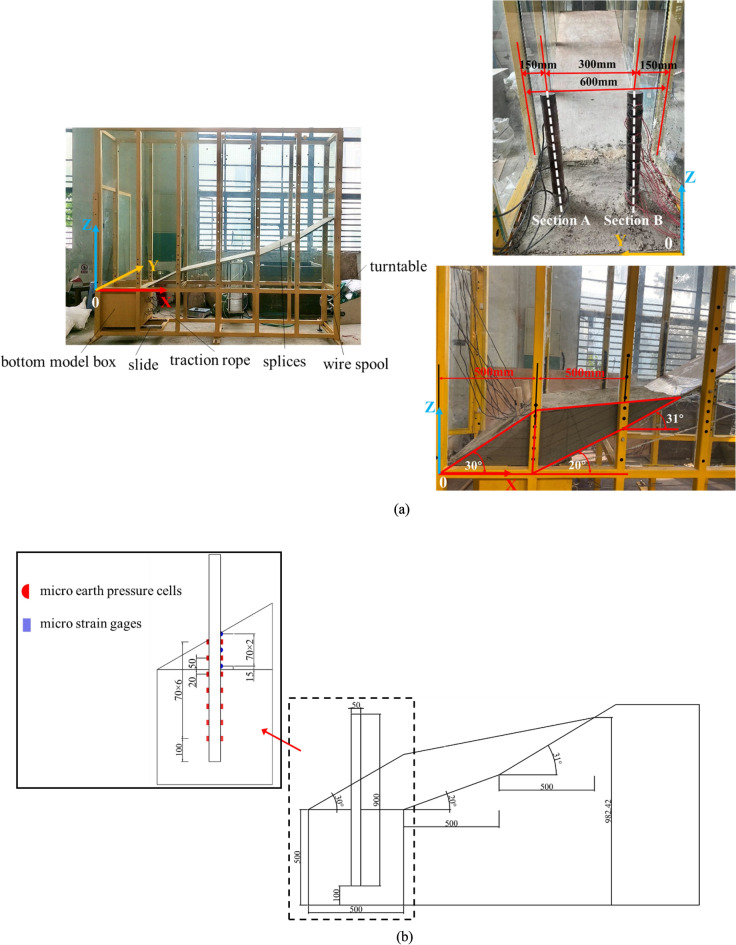
Experimental setup. (**a**) The model test device and installation diagram. (**b**) The view of Section A and B, pressure cells and strain gages positions around pile.

The model box includes scaffold beams, splices, slide bed in bottom model box et al, splices were used to simulate sliding surface as shown in Figure 2a. The dimensions of model box and the bottom box in this study were 600 mm (thickness) × 3000 mm (width) × 2500 mm (height) and 600 mm (thickness) × 500 mm (width) × 500 mm (height). This paper mainly analyzes the soil damage characteristics between the two piles and the stress law of the pile. Due to the pile spacing is 300 mm, the influence range of a single pile is within 150 mm around the pile. Therefore, the distance between the boundary and the center of these two piles in the model test is 150 mm. In addition, the model test assumes that the pile bottom is fixed-end, so the displacement, bending moment and shearing force of pile bottom are zero, the distance between pile and the side boundary of the model box is 15 cm and the distance between the pile bottom and the model box bottom is 10 cm, as described in Figure 2a. As shown in Figure 2b, which is the view of Section A and B in Figure 2a, and the micro pressure cells and micro strain gages positions around pile.

In the process of landslide deformation model test, the deformation is simulated by using artificial loading or hydraulic device to apply soil thrust. The slope model takes a long time to produce deformation, and the labor cost is high with slow change of soil behind the pile and difficult to observe. Moreover, the operation of this method is complicated, because it needs to load on the top of the slope, requires high mechanical properties of the device and the cost is very high. In this paper, it is considered that the landslide deformation behind the pile is entire. The soil mass behind the pile will be compressed and produce force when it encounters the moving pile, which was used to replace the traditional method that reflect the process of landslide deformation. Based on these, in the test of this paper, the pulley is used to slowly pull the bottom model box of the slide bed, making the slip mass and the slide bed produce relative displacement, so as to simulate the landslide displacement. The compression of soil behind the pile makes it produce a certain landslide thrust on the pile, thus changing the force and deformation of the pile.

### The data acquisition device

**Figure 3:**
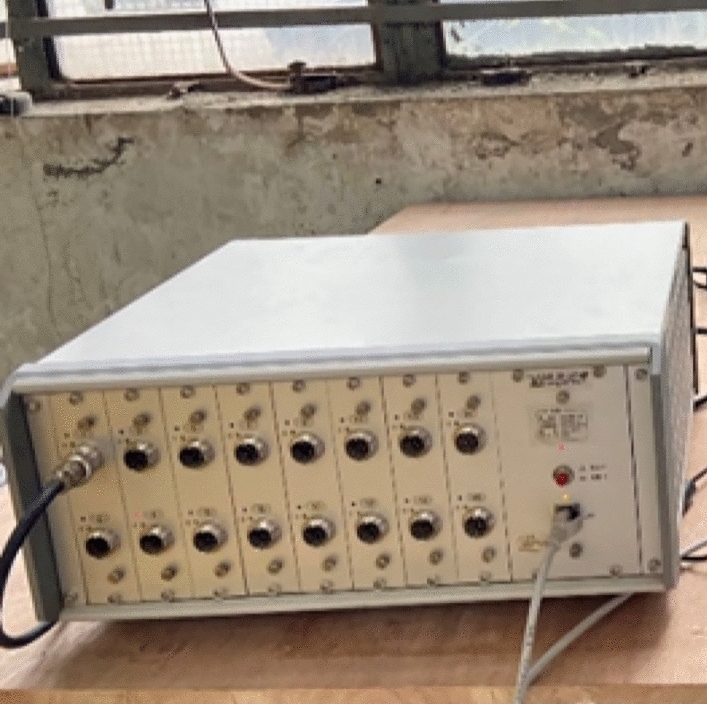
The data acquisition device.

**Figure 4:**
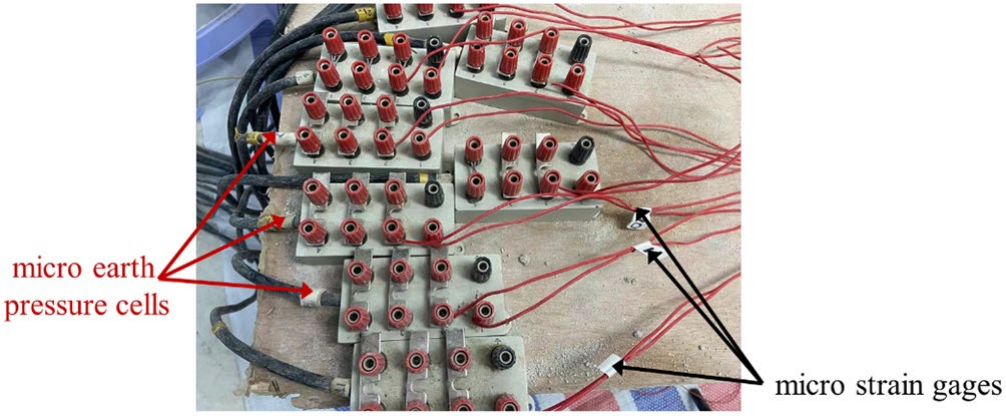
Diagram of connection of monitoring device.

**Figure 5:**
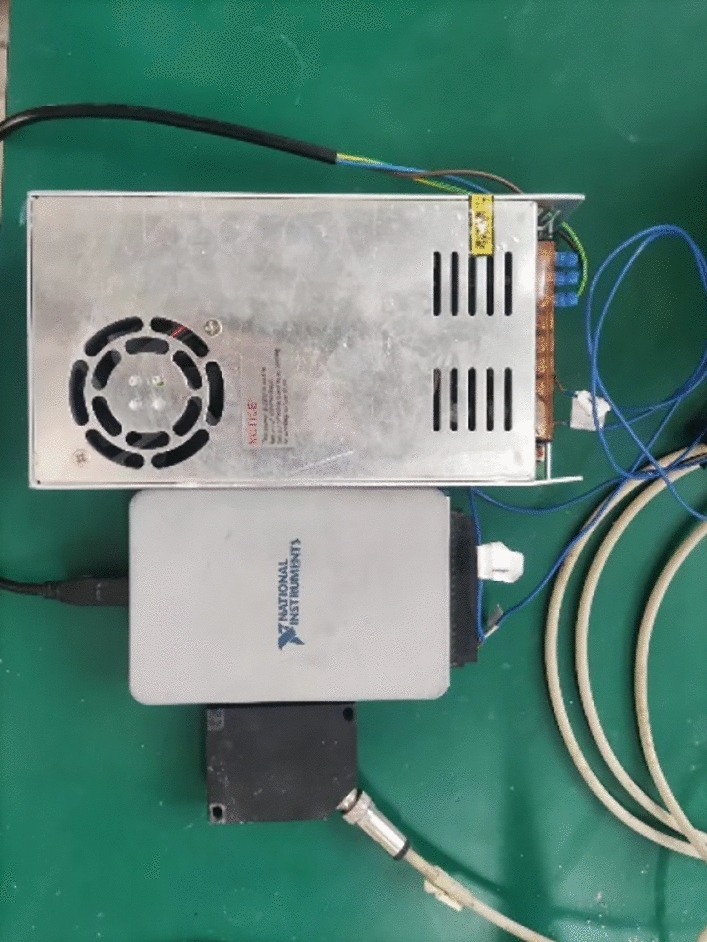
The laser displacement sensor.

The laser displacement sensor, strain acquisition device, micro earth pressure cells have composed the data acquisition device, which was described in Figure 3. The BSF120-80AA strain gages were used to collect the strain of pile side, which could be used to calculated the bending moment of pile, meanwhile the BW micro earth pressure cells were adopted to get the soil resistance. The strain gages and the micro earth pressure cells were described in Figure 4. The laser displacement sensor was shown in Figure 5 is the 1402 laser displacement sensor produced by American Micro Epsilon, which is used to measure the displacement of the bottom model box, that is, the compression of the soil behind the pile.

### Load scheme

The loading system of the model is the displacement of bottom model box in Figure 2. The bottom model box in the slide bed was connected by the rotary plate, and the rotary plate was slowly rotated to make the bottom model box move slowly to the other side, so that the soil compression behind the pile generated pressure on the pile. The displacement of the bottom model box was recorded by laser displacement sensor, the maximum displacement generated by pulling the bottom model box is 17 cm, in which the bottom model box is stabilized for 15 min with every 1cm pulled. The sensor records the earth pressure behind the pile and pile strain, and stops after pulling it to 17 cm. The relationship between the distance of the bottom model box and the force of the pile foundation under different pile diameters, meanwhile the influence of the embedded segment stiffness on the force of the pile was also considered. Table 4 shows the model test conditions.

**Table 4:** The specific loading scheme of model test.

Test number	Pile diameter R (cm)	Deformation of landslide L (cm)	Modulus of slide bed E_e_ (MPa)
1	5	0–17 cm	10
2	5	0–17 cm	20
3	6	0–17 cm	10
4	6	0–17 cm	20

In order to study the rule of pile deformation caused by the instability of landslide surface, the surface of the wooden pile was roughened to simulate pile-soil contact, and the pile displacement from 1 to 17 cm can be regarded as the different deformation of slip mass in this study, the pressure sensors and strain acquisition system can measure the pile internal forces. Moreover, piles with diameters of 5 cm and 6 cm with the same pile length (90 cm) were selected to compare the differences of pile internal force between these two piles’ diameter. For further study, the modulus of slide bed was changed in the test, which can help to preliminary analysis how the supporting layer effect the internal force of pile.

## Results and discussion

### The analysis of pile force and strain in the process of loading

**Figure 6:**
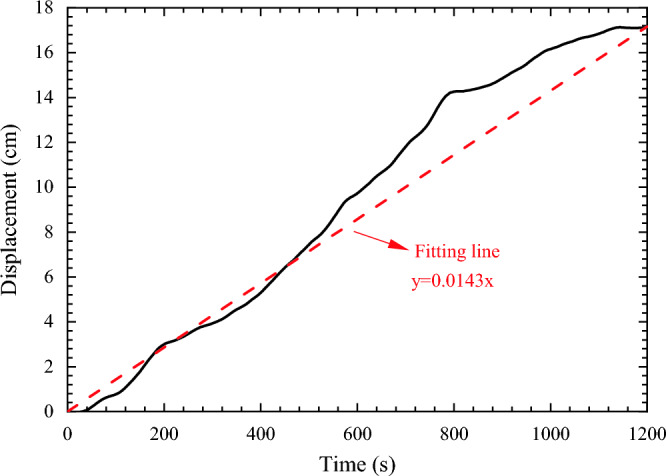
The curve of time and displacement in the process of displacement loading.

In this study, the loading method of displacement control was used, and this paper adopts the way of slowly rotating the turntable to realize the loading of displacement, to ensure that the displacement loading is uniform as far as possible, so that the curve of displacement and time is performed as Figure 6 according to the results of laser displacement sensor, which showed as a line according to the figure that means the velocity in the process of loading could be regard as constant. The velocity is about 0.0143 cm/s as shown in Figure 6.

**Figure 7:**
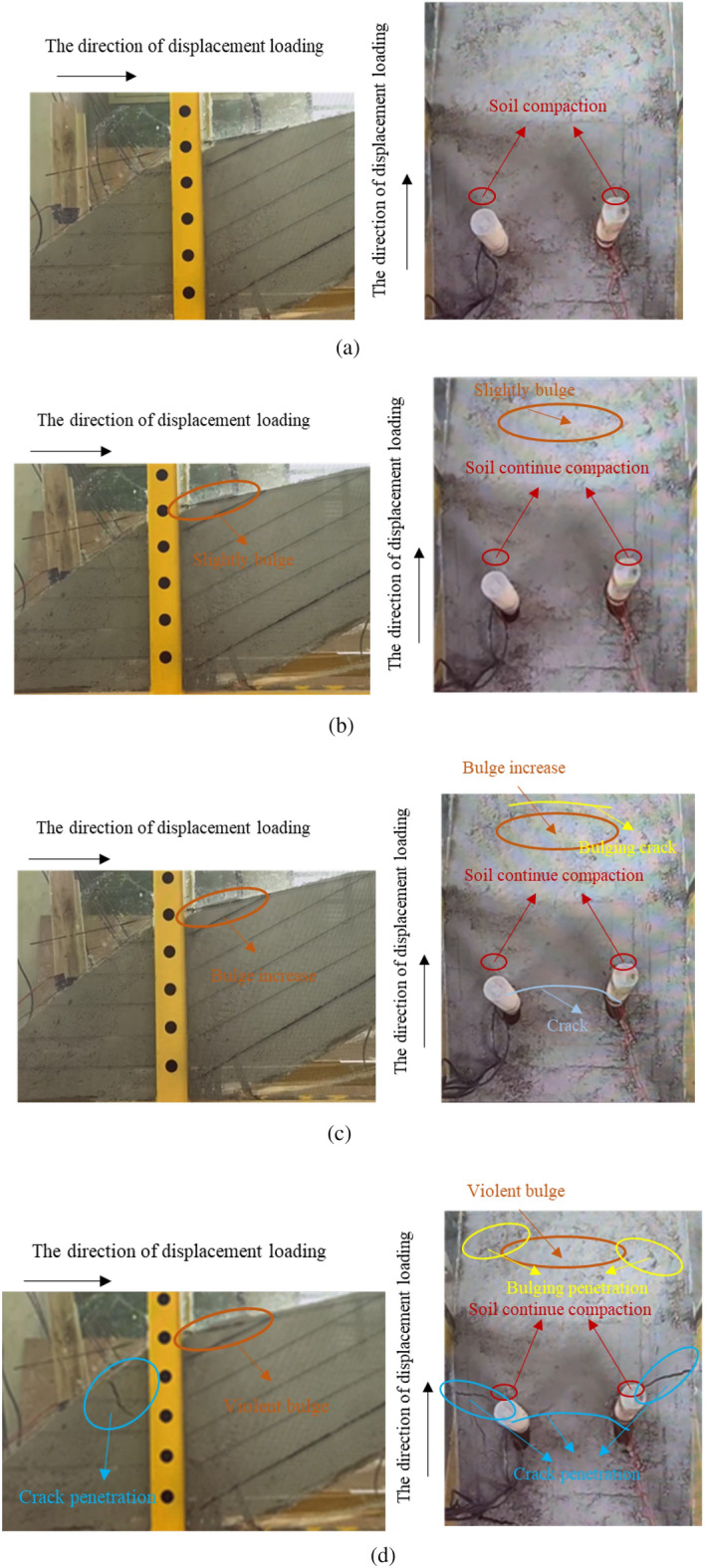
The deformation processes of soil during the model test. (**a**) The initial soil compaction in model test. (**b**) The soil continue compaction in model test. (**c**) The crack growth in model test. (**d**) The soil failure in the model test.

The macroscopic test phenomenon of landslide and pile can reveal the deformation characteristics of landslide and pile in the process of landslide deformation. As shown in Figure 7, at the first stage, the soil behind the pile is slowly compacted, cause the small deformation between the soil and the piles, as described in Figure 7a. In the second stage, the soil compaction behind the pile becomes more significant, generates slightly bulge and cracks according to Figure 7b. With the deformation of landslide increasing, the width of cracks gradually increases at the bulge position of the soil behind the pile as shown in Figure 7c. Finally, there is obvious bulge behind the pile and the soil is destroyed. Some phenomena like the boundary cracks extend down significantly, transverse cracks appear between the piles, arched cracks form between the two piles toward the back, and semi-arched cracks appear symmetrically between the two piles and the boundary are becoming more obvious.

**Figure 8:**
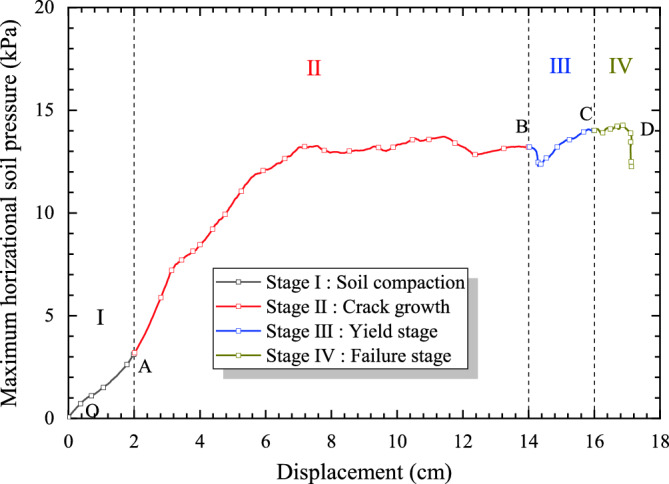
Displacement-stress curve in model test.

**Figure 9:**
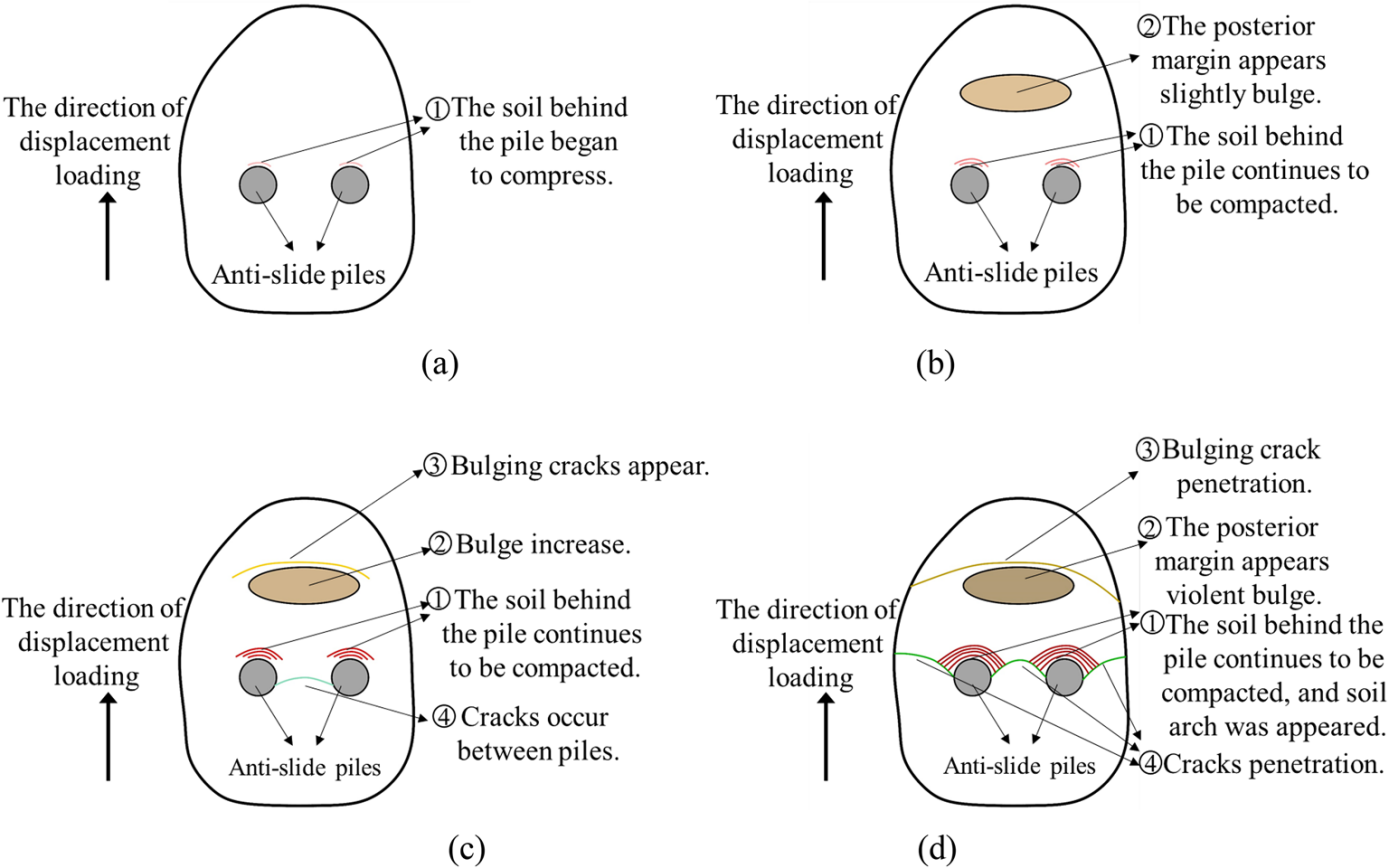
Evolution process and stage division of model test. (**a**) Stage I: soil compaction. (**b**) Stage II: crack growth. (**c**) Stage III: yield stage. (**d**) Stage IV: failure stage.

According to the phenomenon of model tests, the curve of displacement and maximum soil pressure behind pile was obtained as shown in Figure 8. The evolution process of landslide deformation and pile system could be divided into four stages: the Stage I is soil compaction (the curve OA, and the displacement is 0–2 cm), Stage II is cracks growth (the curve AB, and the displacement is 2–14 cm), Stage III is yield stage (the curve BC, and the displacement is 14–16 cm), the Stage IV is failure stage (the curve CD, and the displacement is 16–17 cm). The evolution model of these four stages is shown in Figure 9. The specific description of each stage is as follows:

According to the phenomenon of model tests, the curve of displacement and maximum soil pressure behind pile was obtained as shown in Figure 8. The evolution process of landslide deformation and pile system could be divided into four stages: the Stage I is soil compaction (the curve OA, and the displacement is 0–2 cm), Stage II is cracks growth (the curve AB, and the displacement is 2–14 cm), Stage III is yield stage (the curve BC, and the displacement is 14–16 cm), the Stage IV is failure stage (the curve CD, and the displacement is 16–17 cm). The evolution model of these four stages is shown in Figure 9. The specific description of each stage is as follows:

1. StageI: soil compaction. As shown in Fig. 9a, this stage is the process of soil compaction behind the pile with slight deformation, and there is no obvious macroscopic deformation phenomenon.
2. Stage II: crack growth. In this stage, the loading process had been past more than half, the soil compaction behind the pile made the slope slip and deform along sliding surface, and a slight bulge occurred in the trailing edge of landslide, as described in Fig. 9b.
3. Stage III: yield stage. According to Fig. 9c, due to the continuous displacement loading, the relative displacement of soil and the pile was obviously growing, which leads to the soil pressure behind the pile increased continuously. Meanwhile, more significant bulge and bulging cracks appeared in the trailing edge of landslide, and some cracks appeared between two piles in this model test.
4. Stage IV: failure stage. As shown in Fig. 9d, the relative deformation of the pile and soil is further intensified so that the horizontal displacement of the pile is obvious. Meanwhile, the bulging crack penetration and violent bulge appears in the trailing edge of landslide, soil arch is appeared and gradually eroded to failure, cracks penetration between the pile and the boundary of test model.

### The analysis of pile force and strain in the process of loading

**Figure 10:**
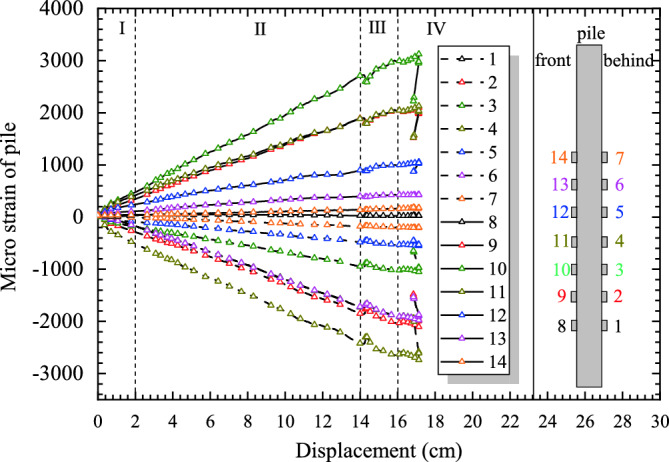
The relationship between displacement loading and micro strain of pile.

Figure 10 shows the variation characteristics of pile strain and loading displacement. The strain data before and after the pile appear symmetrically in pairs. These curves have the same characteristics with four stages as described in Figure 10. The strain growth rate is the highest in the Stage I of the test, slightly decreases in the Stage II of the test, in these two stages is a long compaction process between pile and soil, so that the pile has no obvious deformation. And then the strain growth rate continuous to decreases in the Stage III of the test, the relative displacement of pile and soil gradually increasing due to growing of soil pressure behind pile, thus the strain of the pile became larger. Further the growth rate decreases to zero and finally turns negative in the Stage IV of the test due to the soil failure caused by excessive displacement.

**Figure 11:**
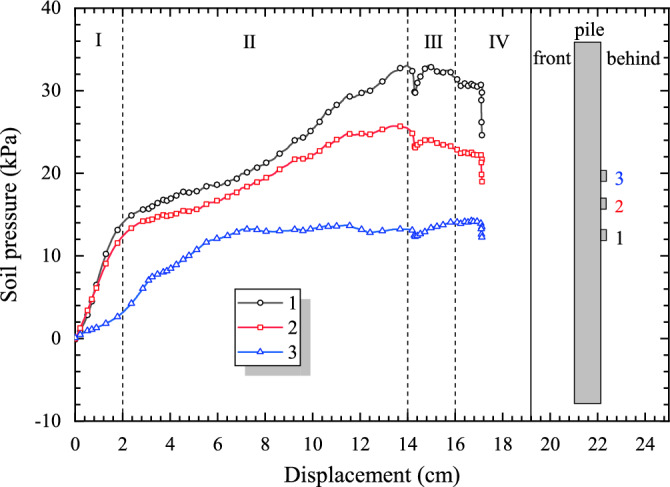
The curve of displacement loading and landslide thrust.

Figure 11 shows the variation characteristics of soil pressure with the changes of loading displacement. It can be found that the curve of each monitoring point on the pile has a similar variation rule, and the curves could also be divided into four stages.

As shown in Figure 11, before the displacement reaches 2 cm is the Stage I of the test, under the action of displacement loading, the landslide surface deformation is small but rapid, so that the soil pressure behind the pile and the soil resistance increase quickly. In the Stage II of the test, when the displacement reaches 14 cm, the model is in the plastic deformation stage, and the soil pressure keeps growing at a constant speed, and its rate is slightly lower than that in the Stage I. In the Stage III of the test, the growth rate of soil pressure further decreased. In the Stage IV of the test, it has been reached to the failure stage of the test, the growth rate of soil pressure has been reduced to almost zero, and the loading displacement has reached the limit of complete failure. The soil pressure growth rate is the largest in the beginning stage of the test, slightly decreases in the middle stage of the test, further decreases in the middle and later stages of the test, and decreases to zero in the final stage of the test.

### The soil pressure along the pile in four different stages

Based on the evolutionary stage division method of displacement loading model test proposed in Section *The analysis of pile force and strain in the process of loading*, the critical points of the four stages are selected as reference points, when the displacement of pile are 2 cm, 14 cm, 16 cm and 17 cm. And the changing rules of soil pressure at different stages are analyzed, as well as the effects of pile diameter and sliding bed strength on soil pressure.

**Figure 12:**
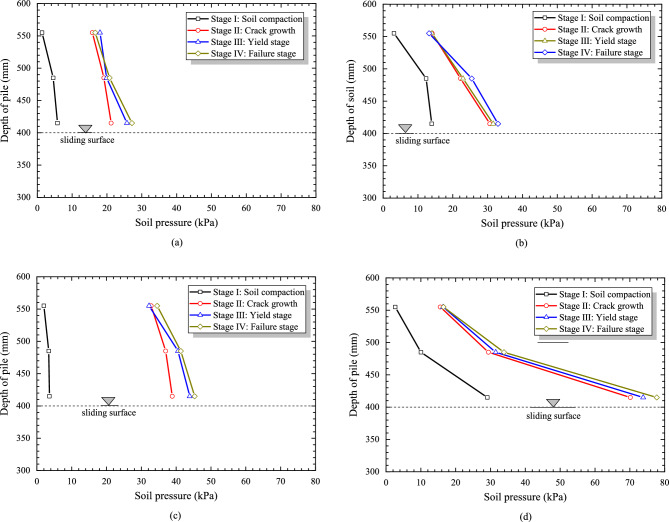
The curves of horizontal soil pressure under different conditions. (**a**) R = 5 cm, Ee = 10 MPa, (**b**) R = 5 cm, Ee = 20 MPa, (**c**) R = 6 cm, Ee = 10 MPa, (**d**) R = 6 cm, Ee = 20 MPa.

According to data from the results of soil pressure cells, Figure 12a–d show the distribution of soil pressure above the sliding surface. The soil pressure increases while closer to the slide surface, and the soil pressure presents an overall triangular distribution along the pile. When the pile diameter is 5 cm and 6 cm respectively, the soil pressure distribution pattern is similar, indicating that the pile diameter is not the main factor affecting the soil pressure distribution. When modulus of the slide bed is 10 MPa and 20 MPa respectively, the soil pressure distribution pattern is also slightly different, but the greater modulus, the larger change rate of soil pressure.

Moreover, as shown in Figure 12a–d, the ratio of the maximum soil pressure growth from the Stage I to the Stage II to the total maximum soil pressure growth in these four Stages is 72.07%, 87.55%, 83.83% and 84.48%, respectively, both exceeding 70% of the total growth in the whole stage. Therefore, it is best to effectively monitor and control the landslide in the initial soil compression stage that in Stage I.

### Bending moment of pile

**Figure 13:**
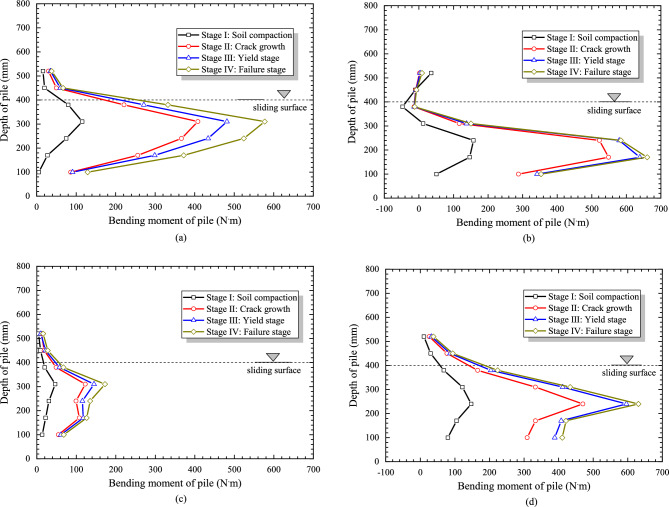
The curves of bending moment of pile under different conditions. (**a**) R = 5 cm, Ee = 10 MPa, (b) R = 5 cm, Ee = 20 MPa, (c) R = 6 cm, Ee = 10 MPa, (d) R = 6 cm, Ee = 20 MPa.

Figure 13a–d are the bending moment distribution diagrams along the pile. According to the above calculation and processing of strain data obtained from the model test, a total of four groups of pile bending moment distribution figures are obtained when the displacement of pile is 2 cm, 14 cm, 16 cm and 17 cm.

According to Figure 13a, the maximum value of bending moment appears at about 100 mm below the sliding surface, and with the increase of loading displacement, the maximum bending moment also increases. The curves first increase and then decrease with the increase of loading displacement, which present parabola distribution rule.

As can be seen from Figure 13a–d, all curves are parabolas. And the pile bending moment distribution is similar when the pile diameter is equal to 5cm and 6cm, which means that the pile diameter of pile is not the main factor affecting the distribution of bending moment. Due to the modulus of slide bed is higher, the maximum value of bending moment is larger, and the position of the maximum bending moment is closer to the bottom of pile.

Moreover, as shown in Figure 13a–d, the ratio of the maximum bending moment growth from the Stage I to the Stage II to the total maximum bending moment growth in these four stages is 63.40%, 77.78%, 60.42% and 66.80%, respectively, both exceeding 60% of the total growth in the whole stage. Therefore, combined with the variation law of maximum soil pressure, it is best to effectively monitor and control the landslide in the initial soil compression stage that in Stage I.

## Conclusion

In order to study the influence of landslide displacement on pile foundation on slope, analysis the soil failure rule and the deformation characteristics of pile in different stages of landslide deformation, a model test device was independently developed, which is based on the displacement control and can improve the test efficiency. Based on this device, the results including landslide displacement, pile bending moment, and soil pressure were obtained during the model test and the following main conclusions can be drawn:

1. According to the phenomenon of model tests, the evolution process of landslide deformation and pile system could be divided into four stages: the Stage I is soil compaction, Stage II is cracks growth, Stage III is yield stage, the Stage IV is failure stage.
2. Above the sliding surface, the soil pressure behind the pile presents like a triangular distribution and the maximum soil pressure increase with the growing of landslide deformation. Meanwhile, with the increase of pile diameter and sliding bed modulus, the soil pressure increases but has no obvious influence on the distribution rule of soil pressure.
3. The bending moment is distributed in a parabola along the pile. The maximum value of pile bending moment is negatively correlated with pile diameter and positively correlated with the modulus of slide bed.
4. According to the rules of soil pressure behind the pile and bending moment of pile, ratios of the maximum soil pressure and bending moment growth from the Stage I to the Stage II to the total growth in these four stages are both exceeding 60% of the total growth in the whole stage, it is best to effectively monitor and control the landslide in the initial soil compression stage that in StageI.

## Data Availability

The datasets used and/or analysed during the current study available from the corresponding author on reasonable request.
